# Do submerged roots hinder orthodontic treatment or the use of implants?

**DOI:** 10.1590/2177-6709.27.5.e22ins5

**Published:** 2023-01-06

**Authors:** Alberto CONSOLARO, Dario A. Oliveira MIRANDA, Renata Bianco CONSOLARO, Mauricio de Almeida CARDOSO

**Affiliations:** 1 Universidade de São Paulo, Faculdade de Odontologia de Bauru (Bauru/SP, Brazil).; 2 Universidade de São Paulo, Faculdade de Odontologia de Ribeirão Preto, Programa de Pós-graduação em Odontopediatria (Ribeirão Preto/SP, Brazil).; 3 Universidade Estadual de Feira de Santana, Departamento de Saúde (Feira de Santana/BA, Brazil).; 4 Centro Universitário de Adamantina (Adamantina/SP, Brazil).; 5 Faculdade de Medicina e Odontologia São Leopoldo Mandic, Programa de Pós-graduação em Ortodontia (Campinas/SP, Brazil).

**Keywords:** Submerged root, Residual root, Diagnostic criteria, Stages of evolution, Orthodontic movement, Osseointegrated implants

## Abstract

**Introduction:**

In clinical practice, submerged roots are found with high frequency, and their presence can change the planning of dental movements and implant placement.

**Objectives:**

To provide explanations of possible developments in the area involved, according to the evolutionary stage of the process, at the time of diagnosis.

**Discussion:**

After atrophy of the periodontal ligament and epithelial remnants of Malassez, ankylosis of the bone with the submerged root occurs, and initiates a process of replacement resorption. Until this process reaches the most advanced stage, this area represents an increased “bone” density, and if some care is not taken, this can generate resorption problems in the tooth to be moved. Whereas implants can be placed, despite the presence of the submerged root, irrespective of the stage of evolution.

**Conclusion:**

It is natural for the onset of alveolodental ankylosis and tooth replacement resorption to occur in submerged roots, and its stage of evolution will be decisive in the approach to be adopted in clinical planning.

## RESUMO

**Introdução:**

A frequência, na prática clínica, das raízes submersas é elevada, e sua presença pode modificar o planejamento de movimentações osteodentárias e da instalação de implantes.

**Objetivo:**

Apresentar as explicações das possíveis evoluções na área envolvida, de acordo com o estágio evolutivo do processo, no momento do diagnóstico.

**Discussão:**

Depois da atrofia do ligamento periodontal e dos restos epiteliais de Malassez, o osso anquilosa-se com a raiz submersa, e se inicia a reabsorção por substituição. Até se chegar ao estágio mais avançado, essa área representa uma densidade “óssea” aumentada, o que pode gerar problemas reabsortivos no dente a ser movimentado, caso alguns cuidados não sejam tomados. Por outro lado, os implantes podem ser aplicados, apesar da presença da raiz submersa, independentemente da fase evolutiva.

**Conclusão:**

É natural que, em raízes submersas, se instale a anquilose alveolodentária e a reabsorção dentária por substituição, e o seu estágio evolutivo será determinante na conduta a ser adotada no planejamento clínico.

## INTRODUCTION

In clinical documentation, images of the jaws of dozens of patients are obtained on a daily basis, and it is very common to find roots submerged in the bone. They may be intact and structurally preserved or at an advanced stage of replacement resorption, after being ankylosed. 

**Figure 1:**
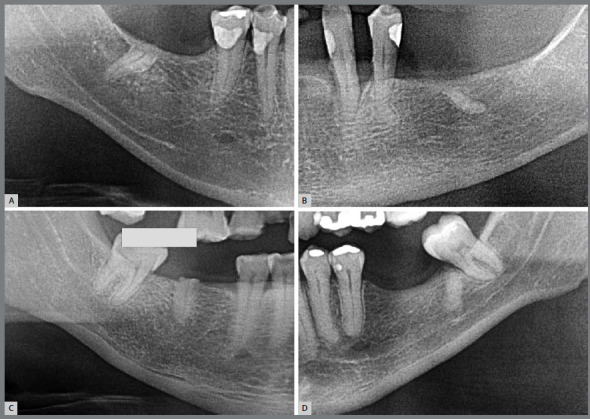
Submerged roots, in the Early Phase of Evolution (**A** and B) and in the Intermediate Phase (**C** and D). After the analysis by panoramic radiography, a more detailed tomographic evaluation is suggested, for control and planning of the case.

In the images, the submerged roots are covered by bone and oral mucosa and almost always, they are not associated with any pathological process, such as periapical lesions, cysts or odontogenic tumors (Fig 1). Submerged roots may appear accidentally or intentionally, after some therapeutic surgical technique has been applied. Submerged roots are characterized into the following types:

a) Apical fragments and those resulting from fractures during tooth extractions.
b) Fragments that persist after accidents involving teeth.
c) After decoronation and its indications^1^ ^,^ ^2^ (third molars in positions with risk of sequelae, hypercementosis, ankylosis and replacement resorption, fractures etc.).
d) After decoronation followed by placement of implants.^1^ ^,^ ^2^
e) After performing techniques involving preservation of tooth structure in periodontal surgeries for esthetic and functional purposes, such as the Socket Shield.

Whereas the term “residual roots” should be restricted to the roots that of which the crowns were destroyed by caries disease or in dental trauma. Generally, they are roots contaminated with pulp necrosis and are surrounded by chronic periodontal disease, as they are exposed to the oral environment and its microbiota.

The term “root remnants” indicates their left over portions that remain and persist, and can be used to describe situations of submerged and residual roots, without distinguishing between them when the distinction between the two terms no longer needs to be so precise.

## MECHANISM: LIGAMENT, RESTS OF MALASSEZ AND EGF ARE DIMINISHED

Over the course of many years without masticatory function, the periodontal ligament-with a thickness of 0.25 mm, the same as a sheet of paper-gradually becomes thinner. In this atrophic process, the epithelial rests of Malassez, which are organized like the net of a basketball hoop with the root inside it, also decrease, thereby increasing the chance of developing a fasciculated alveolar bone in a process of constant remodeling, eventually approaching and touching on the root.^3^ ^,^ ^4^

The epithelial rests of Malassez is the portion that maintains the distance between the tooth and the fasciculated bone, by releasing a mediator, the epidermal growth factor (EGF). EGF constantly stimulates the clasts and cells associated to promote resorption of the periodontal or bony surface of the alveolus. The epithelial rests of Malassez have several physiological functions in the periodontal ligament, with their main function being to serve as the guardian of periodontal space. Therefore, the tooth does not undergo remodeling as is the case of bone, and its roots are preserved.

This atrophy occurs in the periodontal ligaments and in the pericoronal follicles of unerupted teeth that persist in this way for many years. This atrophy occurs in the periodontal ligament of the submerged roots, because, without masticatory function and load, ankylosis and replacement resorption that occur naturally, almost inevitably follow.

## TIME, IMAGES AND STAGES OF EVOLUTION

Once the onset of alveolodental ankylosis has occurred in an area, it gradually spreads, remodels the adjacent bone and the joined tooth, and the cementum and dentin will be replaced by bone. This bone process, known as bone remodeling, eventually replaces the entire root with bone tissue that has all the normal physicochemical and functional characteristics.

Remodeling of all bones is completed every 5 to 10 years, representing a relatively slow gradual and sequential process.^5^ For this process to occur in the jaws and consume a submerged root, logically, it takes several years. Depending on the image detected in radiographs and/or tomography, the period of time of the process that has already elapsed can be classified into the initial, medium or advanced stages.

**Figure 2: f2:**
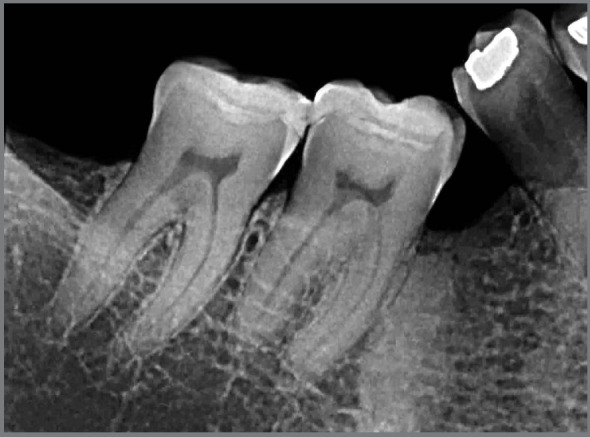
Submerged root, in Early Phase of Evolution, when it still shows traces of periodontal space on most surfaces, with areas of alveolodental ankylosis and replacement tooth resorption.

**Figure 3: f3:**
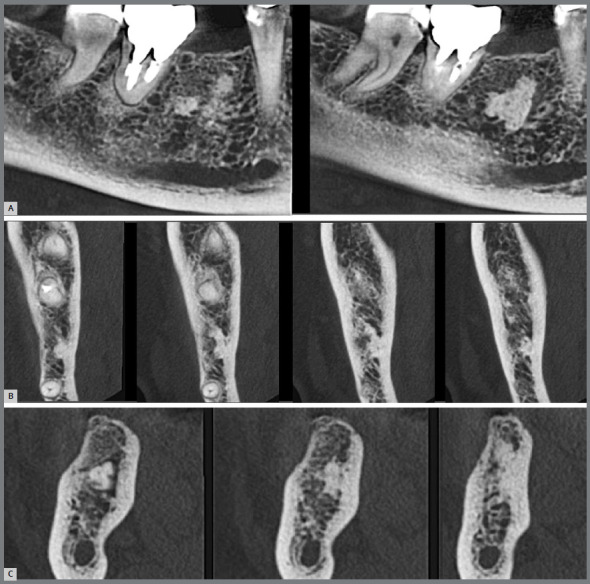
Tomographic images of two contiguous submerged roots, in the Intermediate Phase of Evolution, still showing punctual presence of periodontal space, areas with alveolodental ankylosis and replacement tooth resorption on the majority of surfaces.

**Figure 4:**
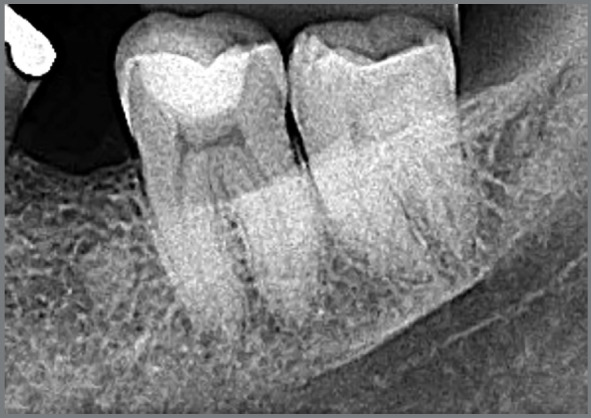
Submerged root in the Intermediate to Advanced Phase of Evolution, still showing punctual presence of periodontal space, areas with alveolodental ankylosis and replacement tooth resorption on the majority of surfaces.

The phases of evolution - the process of disappearance of the submerged root can consist of three evolutionary phases:

1) **Early:** when most of the periodontal space has still been maintained in the images, and only a few small areas have integrated directly with the neighboring bone (Figs 1 and 2).
2) **Intermediate:** when there are areas of periodontal space that have been maintained, but it does not affect the entire extension of the root, which already shows more extensive areas that have integrated with the bone and its trabeculate (Figs 1 and 3).
3) **Advanced:** when there are no longer any areas with preserved periodontal space delimited on the root surfaces, the bone and root remnants become mixed and form part of the normal trabeculae of the region, but an area of sclerosis, suggestive of old root, can still be observed at the site (Fig 4).

## THE PULP AND DIAGNOSTIC CRITERIA OF SUBMERGED ROOTS

The main concern is whether the pulp space contains canal microbiota or not. The submerged roots were sectioned by means of:

a) operating instruments under aseptic conditions or,
b) were ruptured during dental trauma to which they were submitted, and were covered by a blood clot, in an equally aseptic environment.

If the submerged root were exposed to the oral environment or if the environment where it remained was not aseptic, there will be chronic periapical lesions, with or without associated fistula. In practice, this contaminated tooth root assumes the role and consequences of a residual root, with pulp necrosis and microbiota in the canal, as conceptualized in the initial part of this text. As a priority, residual roots should be surgically removed.

For the roots to be diagnosed as submerged in the imaging exams, they must meet the following criteria:

1. There must be no communication with the oral environment.
2. Covered by normal mucosa.
3. No other associated lesions, including fistulas that indicate contamination.
4. Without any symptoms.

The dental pulp of these roots diagnosed as submerged may be found in two situations, namely:

a) The pulp remained vital and was repaired, in the area of the section-as in the areas of pulpotomies cuts and conservative pulp treatments.
b) In sectioning of the pulp and in sudden surgical movements or dental trauma, the apical vessels ruptured and the pulp suffered aseptic necrosis, coagulating its proteins. This aseptic necrosis also occurs in teeth traumatized by concussion, without any symptoms; and coronal darkening is the issue that leads to the patient’s complaint.

Submerged roots are not contaminated, as they do not have any canal microbiota within their structures. Alveolodental ankylosis and replacement resorption will gradually reabsorb them and replace them with bone. If the pulp is necrotic by coagulation, these pulp fragments will be phagocytosed by macrophages and processed as the resorption process progresses. If the pulp has maintained vitality, the pulp tissue will gradually undergo metaplasia to medullary fibrous tissue, and the odontoblasts will die by apoptosis.

## SUBMERGED ROOTS AND ORTHODONTIC MOVEMENT

If there is movement of a tooth to the site when the root is still in the intermediate or advanced phase of evolution, there will be a “bone” density (Figs 3 and 4) that may lead to a considerable risk of lateral root resorption, as if it were an area of idiopathic osseous osteosclerosis or a focal sclerosing osteitis.^6^

In this situation, if you assume the risks and choose to move a tooth towards these denser areas, you should reduce the intensity of the force by between 20 and 30%, and prioritize the distribution of forces along the root of the tooth. 

Due to the loss of elasticity of the bone as a result of the greater density in these areas, the concentration of forces tends to be greater if the usual forces are applied, as there will be no partial dissipation of forces. In other words, there will be 100% action of the force applied at the site, which may generate local root resorption.

A 20 to 30% reduction in forces applied will reduce the effective force to 70 to 80%, allowing for movement through the area without significant root resorption. It may be possible to move the tooth a little more slowly, but safely. 

If the submerged root were in the early stage (Fig 2), the best solution for the patient is to wait until the intermediate or advanced stage is reached. If necessary and there is no time to wait, the submerged root should be surgically removed, before dental movement is induced.

## SUBMERGED ROOTS AND OSSEOINTEGRATED IMPLANTS

In the decoronation technique,^1^ ^,^ ^2^ the tooth in ankylosis and replacement resorption can be considered normal bone as regards its biology and physiology. It can be a site for the placement of osseointegrated implants, without compromising osseointegration. In planning, when opting to apply implants in areas with a submerged root, irrespective of the three phases of evolution, this offers the same possibilities, by following the same care and recommendations (Figs 1 to [Fig f2] [Fig f3] 4).

## FINAL CONSIDERATIONS

1. In submerged roots, if there is no contamination of the area, the natural tendency is for alveolodental ankylosis to occur, followed by replacement root resorption. Over the course of years, the root and its image tend to disappear.
2. From an orthodontic point of view, when submerged root replacement resorption is in the intermediate and advanced stages, the bone at the site should be considered as if it had idiopathic osseous osteosclerosis or focal sclerosing osteitis, making it necessary to reduce the intensity of the forces, as described.
3. If the root is at an early stage of resorption, the risk of localized root resorption of the tooth to be moved increases, and the recommendation is to wait for progression to the following stages of evolution of the submerged roots. If, however, there is little time to wait, the option is its surgical removal.
4. Bone with submerged roots undergoing replacement resorption, irrespective of the stage of evolution in which it is, can be considered biologically normal and able to receive osseointegrated implants.
